# Local Epidemiology of Nosocomial *Staphylococcus aureus* Infection in a Nigerian University Teaching Hospital

**DOI:** 10.3390/antibiotics11101372

**Published:** 2022-10-07

**Authors:** Adeniran Adeyanju, Frieder Schaumburg, Adedeji Onayade, Akinyele Akinyoola, Taofeeq Adeyemi, Osaretin Ugbo, Robin Köck, Yemisi Amusa, Oladejo Lawal, Temilade Adeyanju, Nkem Torimiro, David Akinpelu, Deboye Kolawole, Christian Kohler, Karsten Becker

**Affiliations:** 1 Department of Microbiology, Obafemi Awolowo University, Ile-Ife 220005, Nigeria; 2 Institute of Medical Microbiology, University Hospital Münster, 48149 Münster, Germany; 3 Institute of Public Health, Obafemi Awolowo University, Ile-Ife 220005, Nigeria; 4 Department of Orthopedic Surgery and Traumatology, Obafemi Awolowo University, Ile-Ife 220005, Nigeria; 5 Institute of Hygiene, University Hospital Münster, 48149 Münster, Germany; 6 Institute of Hygiene, DRK Kliniken Berlin, 14050 Berlin, Germany; 7 Department of Surgery, Obafemi Awolowo University, Ile-Ife 220005, Nigeria; 8 Department of Internal Medicine, University College Hospital, Ibadan 200285, Nigeria; 9 Microbiology Unit, Department of Natural and Applied Science, Crown-Hill University, Ilorin 240105, Nigeria; 10 Friedrich-Loeffler Institute of Medical Microbiology, University Medicine Greifswald, 17489 Greifswald, Germany

**Keywords:** *Staphylococcus aureus*, MRSA, nosocomial infection, surgical patients, skin and soft-tissue infections, Panton-Valentine leukocidin, pyrogenic toxin superantigens, exfoliative toxins, epidermal differentiation inhibitors, *agr*

## Abstract

Population-based studies of *Staphylococcus aureus* contribute to understanding the epidemiology of *S. aureus* infection. We enrolled surgical inpatients admitted to an African tertiary-care hospital in order to prospectively analyze the nosocomial impact of *S. aureus*. Data collection included an active sampling of the anterior nares and infectious foci within 48 h after admission and subsequently when clinically indicated. All *S. aureus* isolates were *spa* and *agr* genotyped. Possession of Panton-Valentine leukocidin (PVL) and other toxin genes was determined. We analyzed antibiotic susceptibility profiles by VITEK 2 systems and verified methicillin-resistant *S. aureus* (MRSA) by *mecA/C* PCR. Among 325 patients, 15.4% carried methicillin-susceptible *S. aureus* (MSSA) at admission, while 3.7% carried MRSA. The incidence densities of nosocomial infections due to MSSA and MRSA were 35.4 and 6.2 infections per 10,000 patient-days, respectively. Among all 47 nosocomial infections, skin and soft-tissue (40.4%) and bones or joints’ (25.5%) infections predominated. Six (12.7%) infection-related *S. aureus* isolates harbored PVL genes including two (4.2%) MRSA: overall, seventeen (36.2%) isolates carried pyrogenic toxin superantigens or other toxin genes. This study illustrates the considerable nosocomial impact of *S. aureus* in a Nigerian University hospital. Furthermore, they indicate a need for effective approaches to curtail nosocomial acquisition of multidrug-resistant *S. aureus*.

## 1. Introduction

*Staphylococcus aureus* infections are one of the leading causes of morbidity and mortality worldwide, and their management is challenged by the emergence of MRSA in the past decades [1]. This opportunistic pathogen causes a plethora of infections, ranging from mild skin and soft-tissue abscesses to potentially life-threatening conditions including, e.g., bacteremia, pneumonia, septic arthritis, osteomyelitis, and toxin-mediated illnesses, resulting in increased antibiotic consumption and healthcare costs [2,3]. Presumably, the risk for *S. aureus* infection depends on both patient-related factors and strain-specific differences [1]. Numerous reports described the changing epidemiology and taxonomy of the *S. aureus* complex and addressed the evolution of hospital-adapted, community-associated, and livestock-originated *S. aureus* clonal lineages, as well as changes in antibiotic resistance [4,5,6,7,8,9,10]. In contrast to developed countries, only a limited number of studies elucidated the nosocomial impact of *S. aureus* acquisition in risk patients in resource-limited settings, particularly in sub-Saharan Africa excluding South Africa [11,12,13]. Therefore, analyzing the association with *S. aureus* infection, of patient-specific risks and comorbidities as well as the ecological reservoirs of locally and regionally circulating *S. aureus* genetic lineages can enhance a more targeted, secondary prevention of *S. aureus* infection. Hence, studying the specific characteristics and molecular epidemiology of *S. aureus* in African countries is needed, because, compared with other continents, Africa has different demographics, such as a lower average life expectancy, higher population growth rates and higher total fertility rates, resulting in a generally younger population [14]. In addition, often less stringent antibiotic use policies both in the community and in hospitals and high rates of anti-staphylococcal drug resistance are reported [13,15]. Moreover, toxin gene-harboring *S. aureus* isolates, in particular, those carrying the PVL-encoding genes, were more prevalent in those strains recovered in studies performed in sub-Saharan African countries [16,17,18,19,20]. Therefore, we aimed (i) to prospectively determine the prevalence of nosocomial *S. aureus* infection in a cohort of *S. aureus* carriers and non-carriers; (ii) to determine risk factors for nosocomial *S. aureus* infection; and (iii) to analyze the contribution of PVL toxin gene-positive *S. aureus*, to nosocomial *S. aureus* infection.

## 2. Results

### 2.1. Baseline Characteristics of the Enrolled Surgical Inpatient Cohort and S. aureus Carriage

**Figure 1:**
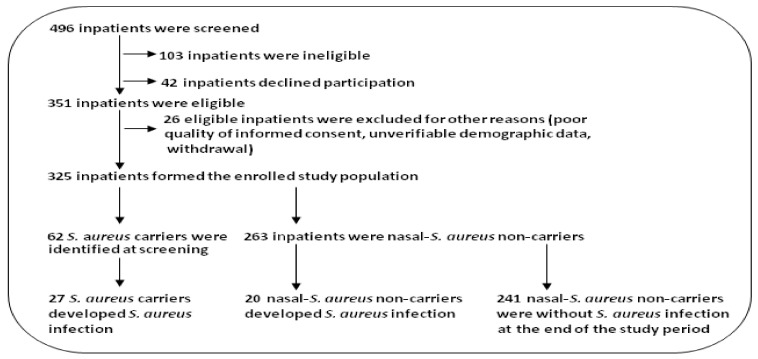
Flow chart of patient screening and passage through the present study.

A total of 325 of 496 surgical inpatients were enrolled (Figure 1). These comprised 182 males and 143 females (mean age ± SD in years: 36.0 ± 18.6 and 37.4 ± 20.3, respectively).

Among the 325 patients, 62 (19.0%) nasal *S. aureus* carriers were identified comprising twelve (3.7%) MRSA and 50 (15.4%) MSSA carriers (the latter including three penicillin-susceptible isolates). Among these 62 carriers, 8 (i.e., 2.5% of all 325 patients) were PVL gene-positive MSSA nasal carriers (16.0% of all MSSA carriers); 3 (i.e., 0.9% of all 325 patients) were PVL gene-positive MRSA nasal carriers (25% of all MRSA carriers). Two-hundred-seventy-nine inpatients (85.8%) had various co-morbidities on admission.

**Figure 2:**
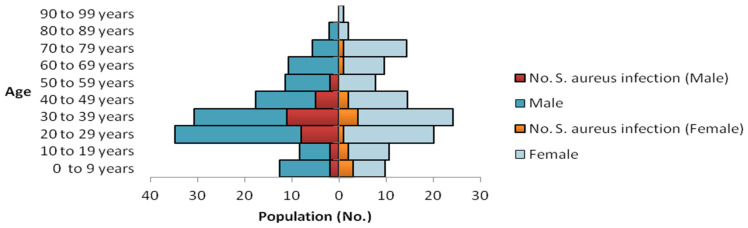
Population age–sex structure of the surgical inpatient cohort (*n* = 325). Superposed within each age group is the age-group specific occurrence of *S. aureus* infection observed within the cohort.

The characteristics of all enrolled inpatients are shown in Table 1. Overall, 47 patients developed *S. aureus* infections. Hence, the incidence density of *S. aureus* infections per 10,000 patient-days was 41.6 (i.e., 35.4 for MSSA and 6.2 for MRSA). The population age–sex structure of the cohort is shown in Figure 2.

### 2.2. Characteristics of Inpatients with Nosocomial S. aureus Infection

In addition to well-known characteristics (recent hospital stay, surgery or antibiotic use), inpatients with nosocomial *S. aureus* infection (*n* = 47) differed from those without *S. aureus* infection with respect to sex and age distribution, the highest odds for *S. aureus* infection was found among males within the age subgroup 30–39 (15/47; 31.9% vs. 56/278; 20.1%, *p* = 0.071, Odds Ratio (OR) = 1.86, 95% confidence interval (CI): 0.87–3.82, Figure 2). While MSSA carriage was clearly associated with nosocomial *S. aureus* infection (24/47; 51.06% vs. 25/278; 8.9%, *p* = 0.0001, OR = 10.56, 95% CI: 4.90–22.6), there was no significant difference in MRSA carriage between the subgroup with *S. aureus* infection and those without *S. aureus* infection (3/47; 6.4% vs. 8/278; 2.8%, *p* = 0.219, OR 2.30, 95% CI: 0.38–10.03). The proportion of patients who received antibiotics was different between the subgroup with and those without *S. aureus* infection (46/47; 97.8% vs. 217/278; 78.1%, *p* = 0.001)). In addition, emergency procedures or two or more surgical procedures were more frequent among patients with nosocomial *S. aureus* infection (11/47; 23.4% vs. 22/278; 7.9% *p* = 0.007, OR 3.06, 95% CI: 1.13-7.63, or 26/47; 55.3% vs. 60/278; 21.6%, *p* = 0.0001, OR 4.50, 95% CI: 2.25–9.01 respectively). Moreover, an association was found with nosocomial *S. aureus* infection among pediatric surgery patients (9/47; 19.1% vs. 10/278; 3.6%, *p* = 0.0002, OR 6.35, 95% CI: 2.12–18.49)). Furthermore, bone and skin and soft-tissue diseases were more frequently observed in patients with *S. aureus* infection (22/47; 46.8% vs. 35/278; 12.6%, *p* = 0.0001, OR 6.11, 95% CI: 2.93–12.59). There was a difference in mean lengths of stay (LOS) ± SD and variance of LOS between the subgroup with (51.36 ± 45.96), and without (31.85 ± 32.67) *S. aureus* infection (*p* = 0.007; for variance: *p* = 0.0006) (Supplementary Table S2).

### 2.3. Antibiotic Resistance Geno- and Phenotypes of Infection-Related S. aureus

**Figure 3:**
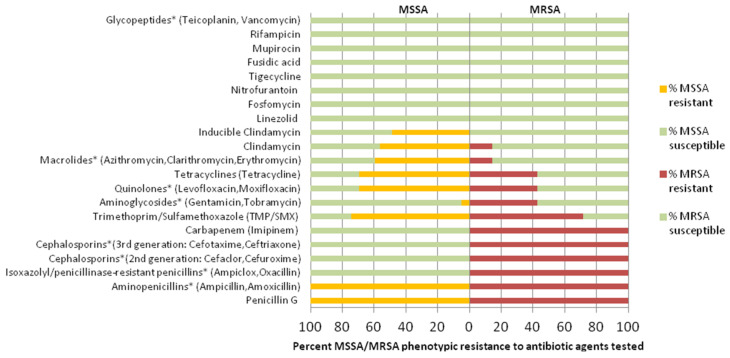
Antibiotic class/agent-specific resistance profiles of nosocomial infection-related MSSA (*n* = 40) and MRSA (*n* = 7) in the form of a gapped population pyramid (MSSA left; MRSA right).

Of all infection-related isolates, 40 *S. aureus* isolates (85.1%) were susceptible to methicillin (including one penicillin-susceptible *S. aureus* isolate) while 7 were MRSA isolates (14.9%, all harboring *mecA*). The antibiotic resistance pattern of MSSA and MRSA isolates is shown (Figure 3).

Among all 47 isolates associated with infections, 61.7% were multidrug-resistant (MDR) according to a consensus definition (either MRSA or resistance to at least 1 antibiotic of ≥3 antibiotic classes) [21]. Table 2 shows resistance profiles for 62 carriage-associated *S. aureus* isolates derived from patients within 48 h after admission, of which 74.2% were MDR.

### 2.4. Distribution of Toxin Genes, Agr Types and Spa/MLST Types

All 47 infection-related *S. aureus* isolates harbored the *nuc* gene. The distribution of toxin-encoding genes and genotypes in these isolates is shown in Table 2. Briefly, all isolates carried the gamma-hemolysin (*hlg*) gene, 15 carried at least one PTSAg or other toxin gene (32.0%), while 13 carried two or more toxin gene combinations (27.6%). Overall, 63.8% (30/47) isolates did not harbor any of the toxin genes tested. The staphylococcal enterotoxin A (*sea*), B (*seb*), C (*sec*), D (*sed*), D–J (*sed*-*sej*) combination, and E (*see) genes* occurred in 17.0%, 4.2%, 0%, 2.5%, 0% and 0% of the isolates, respectively. Of note, toxic shock syndrome toxin *(tst*) and exfoliative toxin A-, B-, and C-encoding genes were not found in infection-related *S. aureus* isolates. Among the non-classical pyrogenic toxin superantigen genes, *seg* occurred in three (3/47; 6.4%) MSSA isolates (in combination with *sei*). One isolate harboring PVL-encoding genes and *sei* was recovered from an episode of necrotizing fasciitis of the left lower extremity in a 48-year-old female, diabetic patient. *seh* and *sej* were found in 3/47 (6.4%) and 0% of the isolates, respectively. The epidermal differentiation inhibitor gene (*edin*A) was detected in 2/47 (4.2%) isolates, and were isolated from a pediatric patient with pyomyositis and a young adult patient hospitalized due to trauma arising from superficial partial-thickness burn. *luk*F and *luk*S PV genes encoding PVL were detected in six (12.7%) infection-related *S. aureus* comprising two MRSA (Table 2).

*Spa* typing identified thirteen distinct *spa* types among 47 infection-related *S. aureus* isolates (Table 2) with t091 (*n* = 28; 59.6%), t084 (*n* = 4), t355 (*n* = 3), t127 (*n* = 2), and t786 (*n* = 2) occurring more than once. The seven MRSA isolates belonged to t091, t355, t786, t037, and t008. ST7 (*n* = 27; 57.4%), ST15 (*n* = 7; 14.9%), and ST1 (*n* = 3) were predominant among MSSA, while ST88 and ST152 (both *n* = 2) were most frequent among MRSA isolates.

**Table 2:** Pattern analyses of all *S. aureus* isolates according to antibiotic resistance phenotype, toxin possession, and genotype.

Pattern	No. of Isolates	*MDR	MSSA/MRSA	P E N	G E N	M O X	E R Y	C L I	T E T	S X T	V A N	PTSAgs, *luk*S-PV and *luk*F-PV, and EDIN Genes	*spa* Type	MLST	*agr* Group
Infection-related *S. aureus* isolates (*n* = 47)															
A	2	**Y**	**MRSA**	R	S	S	S	S	S	R	S	ND	t786	ST88	IV
B	1	N	MSSA	R	S	S	S	S	R	R	S	*sea*	t084	ST15	II
C	1	N	MSSA	R	S	S	S	S	R	R	S	*sea*, *luk*S-PV and *luk*F-PV	t084	ST15	IV
D	2	N	MSSA	R	S	S	S	S	S	R	S	*sea, luk*S-PV and *luk*F-PV	t084	ST15	II
E	1	Y	MSSA	R	S	S	R	R	R	R	S	*sea*, *luk*S-PV and *luk*F-PV	t091	ST7	III
F	1	**Y**	**MRSA**	R	R	R	S	S	R	R	S	ND	t091	ST7	III
G	26	Y ^#^	MSSA	R	S	R	R	R	R	R	S	ND	t091	ST7	III
						I2	S6	S6	S3	S8					
H	1	N	MSSA	R	S	S	S	S	S	S	S	*sea*, *seh*	t127	ST1	III
I	1	N	MSSA	R	R	S	S	S	R	S	S	*seh*	t127	ST1	IV
J	1	N	MSSA	R	S	S	R	S	S	R	S	*seg-sei*, *edinA*	t2724	ST15	II
K	1	N	MSSA	S	S	S	S	S	S	R	S	ND	t355	ST152	III
L	1	Y	**MRSA**	R	S	S	S	S	S	S	S	*sei*, *luk*S-PV and *luk*F-PV	t355	ST152	III
M	1	**Y**	**MRSA**	R	S	S	S	S	S	S	S	*luk*S-PV and *luk*F-PV	t355	ST152	III
N	1	N	MSSA	R	S	S	S	S	S	R	S	*seg-sei*, *edinA*	t311	ST15	II
O	1	N	MSSA	R	S	S	S	S	S	S	S	*seg-sei*	t2731	ST5	III
P	1	N	MSSA	R	S	S	S	S	S	R	S	*sea*, *seb*	t085	ST1	II
Q	1	Y	MSSA	R	R	S	R	S	R	R	S	*sed*	t064	ST8	II
R	1	**Y**	**MRSA**	R	R	I	R	R	R	R	S	ND	t037	ST241	III
S	1	N	MSSA	R	S	S	S	S	S	R	S	*seh*	t7762	ST1	IV
T	1	**Y**	**MRSA**	R	S	I	S	S	R	R	S	*sea*, *seb*	t008	ST8	III
Carriage-associated *S. aureus* isolates (*n* = 62)															
A	29	Y ^##^	MSSA	R	S	R	R	R	R	R	S	ND	t091	ST7	III
						I2	S3	S5		S8					
B	4	**Y**	**MRSA**	R	S	S	S	S	S	R	S	ND	t786	ST88	IV
C	1	**Y**	**MRSA**	R	S	S	S	S	S	R	S	*sea*, *luk* S-PV and *luk* F-PV	t786	ST88	II
D	2	Y	MSSA	R	S	S	S	S	R1	R1	S	ND	t084	ST15	II
E	1	N	MSSA	S	S	R	S	S	S	R	S	ND	t091	ST7	III
F	1	Y	MSSA	R	S	R	R	R	R	R	S	*sea*, *luk* S-PV and *luk* F-PV	t091	ST7	III
G	2	Y	MSSA	R	R	S	S	S	R	R1	S	*sea*, *seh*, *luk* S-PV and *luk* F-PV	t127	ST1	IV
H	2	**Y**	**MRSA**	R	S	R1	R	S	R	R	S	*seg-sei*, *edin*-A	t311	ST15	II
I	4	Y	MSSA	R	S	S	R	S	S	R1	S	*seg-sei*, *edin*-A	t2724	ST15	II
J	1	N	MSSA	S	S	S	S	S	S	S	S	*seg-sei*	t2724	ST15	II
K	2	**Y**	**MRSA**	R	S	S	S	S	S	S	S	*luk* S-PV and *luk* F-PV	t355	ST152	III
L	2	**Y**	**MRSA**	R	S	S	S	S	R	S1	S	ND	t355	ST152	III
M	2	N	MSSA	R	S	S	S	S	R1	S	S	ND	t355	ST152	III
N	1	N	MSSA	R	S	S	S	S	R	S	S	*sea, seb, sec, luk * S-PV and *luk* F-PV	t064	ST8	III
O	1	**Y**	**MRSA**	R	S	S	R	S	R	R	S	*sea*, *seb*	t064	ST8	III
P	1	N	MSSA	R	S	S	S	S	R	S	S	*luk* S-PV and *luk* F-PV	t4690	ST153	III
Q	2	N	MSSA	R	S	S	S	S	S	R	S	*luk* S-PV and *luk* F-PV	t355	ST152	III
R	1	N	MSSA	R	S	S	S	S	S	R	S	*sea, luk* S-PV and *luk* F-PV	t084	S15	II
S	1	N	MSSA	S	S	S	S	S	R	S	S	*seg-sei*	t091	ST7	II
T	1	Y	MSSA	R	S	R	R	R	R	R	S	*tst*	t091	ST7	III
U	1	Y	MSSA	R	S	R	R	R	R	S	S	ND	t1685	ST7	III

*Multidrug resistant [21]. ^#^ Twenty infection-related *S. aureus* isolates were multidrug resistant. ^##^ Twenty-three carriage-related *S. aureus* isolates were multidrug resistant. Y- Yes; N- No; *spa*, staphylococcal protein A; MSSA, methicillin-susceptible *S. aureus*; MRSA, methicillin resistant *S. aureus;* PTSAg, pyrogenic toxin superantigen; *agr*, accessory gene regulator; MLST, multilocus sequence typing; *hlg*, gamma-hemolysin; *sea*-*sei*, staphylococcal enterotoxin genes A-I; *edinA*, epidermal differentiation inhibitor A gene; *luk*S-PV and *luk*F-PV, Panton-Valentine leucocidin genes; ND, none of the tested virulence genes detected. R-resistant, S-susceptible, I-intermediate PEN penicillin, GEN gentamicin, MOX. moxifloxacin, ERY erythromycin, CLI clindamycin, TET tetracycline, SXT trimethoprim/sulfamethoxazole, VAN vancomycin. Detection of susceptible or resistant variant(s) is (are) indicated. (Numbers indicate variants ≥ 1). Grey row(s) identify genotypes common to infection-related and carriage-related *S. aureus* isolates. Black row indicates putative USA300 ST 8-related, isolate. Mean (median) number of antibiotics to which infection-related *S. aureus* isolates (n = 47) were resistant: 10.5 (10.4). Mean (median) number of antibiotics to which carriage-related *S. aureus* isolates (n = 62) bore resistance: 10.5 (9.67). Total number of antibiotics tested, n = 30. Note: pattern nomenclatures (e.g., A, B, C, etc) are for identification purposes. Pattern nomenclatures do not generally suggest pattern correlation across both isolate collections.

All isolates were *agr*-typeable (*agr* II: *n* = 7, 14.9%; *agr* III: *n* = 35, 74.4%; and *agr* IV: *n* = 5; 10.6%); *agr* I was not detected. While PTSAg gene-carrying *S. aureus* more frequently belonged in *agr* group II (*n* = 5; 41.6%) and *agr* group III (*n* = 4; 28.5%), *edin*A gene-carrying *S. aureus* detected in the present study exclusively belonged to *agr* group II (*n* = 2; 100%) and strictly carried the *seg-sei* gene combination.

PVL-encoding genes frequently belonged to *agr* group III (*n* = 3; 6.4%) and *agr* group II (*n* = 2; 4.2%). MSSA were mostly associated with *agr* group III (77.5%; 31/40) and *agr* group II (15.0%; 6/40), while MRSA predominantly belonged to *agr* group III (57.1%; 4/7) and *agr* group IV (28.5%; 2/7). Table 2 shows a pattern analysis of the characteristics of infection-related and carriage-associated *S. aureus* isolated from surgical inpatients. The pattern analysis revealed 15 and 16 distinct genotypes of infection-related and carriage-associated *S. aureus* strains, respectively (Table 2). Five genotypes (t786/ST88, t084/ST15, t091/ST7, t2724/ST15, and t355/ST152) represented 25.0% and 23.8% of both infection-related and carriage-associated *S. aureus* genotypes, respectively. In general, MLSTs frequently linked to toxin gene possession included ST1, ST5, ST8, ST15, and ST152 whereas ST7 and ST88 rarely possessed toxin genes. In contrast, ST7, ST8, ST15, ST88, and ST241 displayed a high frequency of antibiotic resistance traits compared to ST1 and ST5, which comprised mostly susceptible strains. A notable combination of toxin gene possession and multi-resistance traits was observed in ST8 and ST15. On the level of individual patients, we found that, of 47 *S. aureus* isolates identified from sites of nosocomial infection, 27 (57.4%) shared the same *spa* type as those colonizing the respective patient at (or within 48h after) admission. Among the 27 *spa* types, heterogeneity within *spa* types was apparent in two cases.

### 2.5. Stratification of Patients with Nosocomial S. aureus Infection According to Nasal S. aureus Carriage

Supplementary Table S1 shows the characteristics of patients with *S. aureus* infection stratified by nasal *S. aureus* carriage status at admission. Table 3 summarizes the risk factors observed, and those associated with nosocomial *S. aureus* infection in carriers vs. non-carriers. Overall, the nosocomial *S. aureus* infection rate was 27/62 (43.5%) among patients carrying *S. aureus* within the 48 h after admission vs. 20/263 (7.6%) among *S. aureus* non-carriers (*p* = 0.0001; RP 5.73 99% CI (2.93–11.13)). We observed that *S. aureus* carriers and non-*S. aureus* carriers differed regarding their risk to develop a nosocomial infection. In particular, *S. aureus* carriers with underlying bone disease (*p* = 0.008; RP 4.04 (0.54–19.5)), cardiovascular disease (RP 8.50; 1.54–47.04), or diabetes mellitus (RP: 8.00; 0.72–89.03) had a higher risk for nosocomial *S. aureus* infection compared with non-carriers affected by these comorbidities. In addition, the total number of patient-days was significantly higher among *S. aureus* carriers with *S. aureus* infection than among *S. aureus* non-carriers (*p* = 0.0005; RP: 5.01 (4.58–5.48)), suggesting added healthcare costs for *S. aureus* carriers with nosocomial *S. aureus* infection. Among inpatients who received invasive devices, an intravenous device received intermittently for more than 24 h was associated with a significantly increased risk for *S. aureus* infection in *S. aureus* carriers than non-carriers (*p* = 0.0001; RP 6.00; 1.45–24.82). Similarly, emergency surgery (RP 7.88; 1.32–46.93), or two or more surgeries (RP 3.37; 1.42–7.95) was associated with an increased risk for nosocomial *S. aureus* infection. Overall, the stratification analyses not only confirmed a higher incidence density of nosocomial *S. aureus* infection in *S. aureus* carriers vs. non-carriers (106.7 vs. 23.1 *S. aureus* infections per 10,000 patient days, respectively), it also proportionately revealed risks associated with nosocomial *S. aureus* infection in *S. aureus* carriers versus (vs.) *S. aureus* non-carriers in the setting studied (Table 3).

## 3. Discussion

In this prospective study, we describe the local epidemiology of and risks for nosocomial *S. aureus* infection in a cohort comprising surgical inpatients. Regarding antibiotic resistance profiles, antistaphylococcal benzylpenicillin or aminopenicillin resistance reached 98% and 100% among infection-related isolates in the present study, consistent with findings from neighboring African countries [13]. All MRSA strains displayed, as expected, complete resistance to isoxazolyl penicillins, second and third generation cephalosporins, and carbapenem, in sharp contrast to MSSA strains which tested completely susceptible to these anti-infective agents. Interestingly, we found apparently lower MRSA vs. MSSA resistance (71.4% vs. 74.3%, respectively) to trimethoprim/sulfamethoxazole, a widely used antibiotic in community settings in this region while a similar pattern of MRSA vs. MSSA resistance was displayed to quinolones (42.8% vs. 69.2%), tetracycline (42.8% vs. 69.2%), macrolides (14.3% vs. 59.6%), and clindamycin (14.3% vs. 56.4%) making us think that appropriate combinations of these antibiotics may preserve efficacy against certain MRSA infections. Moreover, high rates of inducible clindamycin resistance seen among MSSA strains (48.7%) suggest that to checkmate the growing threat of clindamycin resistance among MSSA strains (56.4%), clindamycin use, where possible, should be discouraged for treatment of certain MSSA infections. Overall, all MSSA and MRSA strains were completely susceptible to linezolid, fosfomycin, and vancomycin, in line with previous findings [16]. The same was true for nitrofurantoin, fusidic acid, mupirocin, and rifampicin (Figure 3). However, resistances to fusidic acid [22], mupirocin [23], nitrofurantoin [24], rifampicin [25] and vancomycin [24] have been reported in other studies performed in Africa. During the study period, we were able to include 92.3% (325/351) of the eligible patients screened after admission. We found that in this setting, the crude rate of nosocomial *S. aureus* infection was 14.5% (47/325). This was equivalent to an overall incidence density of 41.6 *S. aureus* (MSSA: 35.4 and MRSA: 6.2) infections per 10,000 patient days. This rate is comparable to the findings of a WHO study [26] performed in 14 countries worldwide where, on average, 8.7% of the patients treated in hospitals developed nosocomial infections (due to all causative agents). This rate ranged from 5.0% in North America to 40% in Asia, Latin America, and the Sub-Saharan regions of Africa. Allegranzi et al. found that the prevalence of nosocomial infections was 15.5% in developing countries when pooling data from more than 100 studies performed until 2008 [27]. In our study, surgical-site infection was the most important infection occurring in 5.6% of 341 surgical interventions. Hence, our data are comparable, as *S. aureus* substantially contributes to the burden of healthcare-associated infection, especially in surgical patients, but exceed infection rates found in surgical patients in industrialized settings. For example, in the Netherlands, 40 of 1,980 patients (2%) developed sternal *S. aureus* wound infections after cardiac surgery [28], in Switzerland, 1.06% developed MRSA surgical site infections [29], and in the UK, 4.5% developed *S. aureus* wound infections in a historical cohort in the 1950s [30]. Compared to industrialized settings, one explanation for the high rate of *S. aureus* infection in the present study could be the very long length of hospitalization of the patients included in this study (>30 days). This exceeds by far the averages for patients in industrialized countries (5–6 days) and increases the risk of nosocomial infection irrespective of the causative agent. The LOS observed might be explained by the fact that most surgical patients enrolled in this study (60%; 196/325, Table 1) were hospitalized for trauma surgery; correspondingly, rates of clean-contaminated procedures were high, > 80% in this study (Table 1, Supplementary Figure S1) in contrast to rates seen in industrialized settings (<10%), altogether suggesting increased antibiotic use or resistance and nosocomial burden of infection, as shown. Importantly, we found that the overall risk of nosocomial *S. aureus* infection was not equally distributed among the included patients. We observed a clearly significant difference of the infection risk between *S. aureus* carriers (27/62 (43.5%) or 106.7 infections/10,000 patients-days) vs. *S. aureus* non-carriers (20/263 (7.6%) or 23.1 infections/10,000 patients-days; Table 3). However, it should be borne in mind that it was not the intent of the present study to prove that carriage of *S. aureus* in the nasal cavity, which is known as the principle Habitat of *S. aureus* [31,32,33], caused subsequent *S. aureus* infection. This subject has been addressed in earlier reports [34,35,36]. Rather, owing to the role of nosocomial reservoirs in the epidemiology of infection, we collected prior screening samples from the anterior nares in order to better understand the extent of involvement of this ecological niche in *S. aureus* infection in the hospital setting in view, further aiding risk classification, and separation of endogenous *S. aureus* infection for infection control purposes. Whereas 20 of 263 previously uncolonized patients had at least an episode of *S. aureus* nosocomial infection, 2 of 27 patients colonized before had an infection caused by a strain associated with a *spa* type and phenotypic antibiogram other than the colonizing strain. Regarding the types of nosocomial infections detected, *S. aureus* skin and soft tissue infections’ (SSTIs) isolates predominated (40.4%; 19/47) in this surgical inpatient cohort, mostly associated with multi-resistant MSSA genotypes such as t091 ST7 *agr* III, t786 ST88 *agr* IV, t355 ST152 *agr* III, t127 ST1 *agr* IV. Intriguingly, ST1-, ST7-, and ST88-related *S. aureus* SSTIs have been detected in an Asian healthcare setting [37], signaling inter-regional dissemination. *S. aureus* bone and joint infections (25.5%; 12/47) were also recorded frequently among a subset of patients with a recent history of road traffic-related trauma of the extremities, implying direct implantation or contiguous-focus acquisition. Since *S. aureus* binding to host tissue is an important precedent to infection, we speculate that the observed predominance in the anatomical distribution of *S. aureus* infections may reflect, in part, a gradient of *S. aureus* selectivity for host tissue, i.e., binding avidly to stratified squamous epithelial, or periosteal tissue including bone matrix and collagen, than to columnar or transitional epithelium [38]. An explanation for this discrepancy also could be that most of the surgical-site infections (63.1%, 12/19) occurred among patients admitted with open, traumatic wounds. These are patients with an increased infection risk, because compared to elective procedures, aseptic techniques are more likely to fail, and preoperative decolonization therapies are likely not to be applicable.

Recovery of *S. aureus* from the nares in patients with a previous history of hospitalization suggests that previous hospitalization may predispose to host adaptation, prolonged carrier state, or subsequent infection. In the present study, prior healthcare contact (*p* = 0.0002, RR (95% CI): 3.64 (1.60-7.96)) was associated with patients with *S. aureus* infection (Supplementary Table S2), in line with similar findings elsewhere [39]. In general, we observed a significant risk for *S. aureus* infection in carriers, where the duration of hospitalization was ≤8 weeks (*p* = 0.0001, RR (95% CI): 7.02 (2.08-23.70)), invasive devices were present (*p* = 0.0001, RR 4.16 (2.30–7.50)), emergency surgery (*p* = 0.0001, RR 7.88 (2.02–30.63)) or two or more surgeries (*p* = 0.0001, RR 3.37 (1.75–6.48)) were received, or where comorbid conditions such as bone disease (*p* = 0.008, RR 4.04 (1.22-13.39)) or bone, skin, and soft tissue diseases (*p* = 0.0005, 2.89 (1.51-5.52)) were present (Table 3).

PVL-possessing *S. aureus* strains are mainly associated with deep, often recurrent SSTIs [40], with a plausible but controversial role in virulence. In the present study, PVL toxin genes-positive *S. aureus* was detected more frequently in acute SSTIs (66.7%; 4/6) than in other clinical specimens (33.3%; 2/6). This is consistent with the findings of a previous report [40]. Whilst two of eleven PVL toxin genes-positive *S. aureus* carriers (18.2%; 2/11) developed *S. aureus* skin infections, other PVL toxin genes-positive *S. aureus* infections were observed in non-*S. aureus* carriers. Besides putative transmission of the clones, our observation may also reflect the mobility of PVL phages between *S. aureus* lineages. Of note, when screening for nasal carriage, we detected a PVL-positive MSSA ST 8 isolate in one patient, which had a *spa* repeat pattern (t064: 11-19-12-05-17-34-24-34-22-25) similar to the USA300/ST8 (t008: 11-19-12-21-17-34-24-34-22-25) clone, indicating that a rarely detected *S. aureus* clone with similar characteristics to USA300 is present in sub-Saharan Africa, in the form of aberrant/mutant *spa* types, mostly MSSA [41] whereas most PVL-positive *S. aureus* isolates in this study were associated with *agr* group III (50%; 3/6 for infection-related *S. aureus*, 81.2%; 9/11 for carriage *S. aureus*), which confirms previous findings [42,43] and might suggest that *agr* independently, or together with specific virulent factors, may explain certain clinical features of *S. aureus* infection [43]. Concerning the low occurrence of PVL-positive *S. aureus* infection in PVL toxin genes-positive *S. aureus* carriers, we think that this might either be due to the small number of carriers detected or reflect immunologically modulated protection, already shown for toxic shock toxin (TSST)-producing *S. aureus* carriers [44]. It could also be that altered C5a or CD45 PVL domains on neutrophils [45] are equally important determinants of PVL-positive *S. aureus* infection.

Apart from PVL toxin genes, we found 32.0% PTSAg gene-encoding, infection-related *S. aureus* predominantly in patients with a recent history of trauma. In addition to classical sympathoadrenal and metabolic responses to trauma, the proinflammatory cytokine component may be aggravated by PTSAg-producing *S. aureus* strains [46], with consequent adverse, localized, or systemic sequelae. Further on, we detected EDIN A toxin gene in exclusive association with *seg-sei* genes, in two infection-related *S. aureus* isolates (spa type t2724, t311). EDIN toxins are Ras-homolog-A GTPase-targeting and may interrupt intracellular signaling in *S. aureus*-infected host cells, disrupting molecular switches governing cytoskeletal architecture and other vital cell processes, with important consequences for host cell regulation and survival [47]. Although we observed a low prevalence of EDIN A toxin gene-positive *S. aureus* infection (2/47; 4.2%) and apparent lineage specificity (being limited to ST15), a higher prevalence of *edin* A positive *S. aureus* has been reported elsewhere [48]. In contrast to *edin* A (4.2%), PVL toxin genes (12.7%), and other variably detected PTSAg genes (32.0%), the invariable detection of leukotoxic *hlg* genes in all infection-related (100%; 47/47) and carriage-related (98.3%; 61/62, not shown) *S. aureus* isolates in the present study, might reflect a strong role for *hlg* in molecular patho-mechanistic events preceding *S. aureus* disease. A recent experimental study suggests a direct role for *hlg*AB in the competition for and successful hijacking of atypical chemokine receptor 1 (ACKR1, a G protein-coupled receptor) in the early stages of *S. aureus* disease [49], whereas the cytotoxic effect of the PVL toxin, but not HLG, may be neutralized [50].

Although we aimed for 95% statistical power to detect 50% *S. aureus* infections in *S. aureus* carriers, with one-half difference in prevalence between non-*S. aureus* carriers, since we enrolled less than the required number of patients, a post-hoc power evaluation undertaken to find the actual power of the present study (given *n* = 325, α = 0.01, other design parameters unchanged) showed that the present study had 82.8% power to detect 43.5% *S. aureus* infection among *S. aureus* carriers, with approximately one-half distance in prevalence from non-*S. aureus* carriers. Although we observed a lower (0.435) than assumed (0.50) proportion of *S. aureus* infection among *S. aureus* carriers, we found no significant difference between both proportions using the z test, the z score (0.80) being less than 0.01 and 0.05 values of z (2.58 and 1.96, respectively), thus confirming initial study assumption(s) among *S. aureus* carriers. Given adequate attention to statistical power evaluation, the probable error associated with infection control decisions is known. Since we quantified the effect of classical and perceived risk factors including co-morbidities (Table 3), our findings demonstrate opportunities for *a priori* determination of risk for *S. aureus* infection, but also for evaluation of costs for infection control approaches. Limitations included that many patients received prophylactic broad-spectrum antibiotic therapy. Therefore, the clinical impact of *S. aureus* infection might not have been fully captured. In addition, due to limitations of *spa* typing in resolving genetic differences, an allele-based typing method could further clarify genetic links between spatiotemporally related *S. aureus* strains. Overall, we observed a discrete, non-random occurrence of nosocomial *S. aureus* infection, with a tendency for clustering, especially among those surgical specialties comprising inpatients admitted with comorbidities involving the integumentary or musculoskeletal system.

## 4. Materials and Methods

### 4.1. Study Design and Setting

At the time of the study described here (2012/2013), the study site, the Obafemi Awolowo University Teaching Hospitals Complex in Ile-Ife, was an approximately 500-bed hospital serving an estimated 4,140,228 inhabitants of Osun State, Nigeria. The hospital is a major referral healthcare facility in southwestern Nigeria and provides specialized medical services across various specialties including in internal medicine, obstetrics and gynecology, pediatrics, and surgery. Specialty or subspecialty services from where patients were screened and enrolled are listed in the body of the report (Table 1).

We planned to screen eligible patients for MSSA and MRSA carriage within 48 h after admission in order to be able to delineate the proportion of subsequent *S. aureus* infections attributable to endogenous sources. We simulated effective inpatient sample sizes at different α and β levels, by optimizing for two effect sizes: the effect size of *S. aureus* carriage [16], and secondly, the effect size of *S. aureus* infection. The average ratio of non-*S. aureus* carriers to *S. aureus* carriers (3.1–4.0: 1.0) in this setting was also determined [16,17,18]. The theoretical probability of the prevalence of *S. aureus* infection was deduced from the *S. aureus* carriage rate. PVL toxin gene proportions from selected studies in this region [16,17,18] were pooled to estimate the occurrence of PVL toxin gene-positive *S. aureus* (≥ 0.113 PVL ± 0.063 (95% SE)). Criteria for selection of pooled studies included, performance in a healthcare setting in sub-Saharan Africa, a clearly defined population focus, analysis of carried *S. aureus* strains, and a prospective approach. Assumptions included: (i) a discrete asymptotic distribution of *S. aureus* infection cases, (ii) α = 0.01; β = 0.05, (iii) a one-tailed test (degree of freedom (df) = 1)). We hypothesized that at least 50% of enrolled inpatients who carried *S. aureus* would develop an *S. aureus* infection episode during the same (or later, related) hospital stay, while this risk was diminished arbitrarily by half (i.e., 25%) for non-*S. aureus* carriers, in order to justify the possibility of inadvertent *S. aureus* acquisition. Our assumptions implied an optimal sample comprising 376 inpatients (301 non-*S. aureus* carriers vs. 75 *S. aureus* carriers) for an unmatched cohort design. Critical value of χ^2^: 5.41.

### 4.2. Patients

All inpatients who met the inclusion criteria and who gave written (occasionally, if not literate, oral) informed consent were considered eligible. For each patient, anonymized data were collected at study inception by a review of the medical record including age, gender, occupation, specialty/subspecialty affiliation, date and indication of admission, ward, co-morbid conditions, prior hospitalization, prior antibiotic use, prior surgery, presence of indwelling device(s), presence of any open wounds or potentially infectious foci. Records with missing data were clarified by patient interviews or excluded. McCabe scores were derived as previously described [51]. Subsequent data collection comprised clinical specimens, surgical treatment classification (emergency or elective), length of stay, number of patient-days and antibiotic use (all antibiotics belonging to class J01 of the World Health Organization/Anatomical Therapeutic Chemical Classification (WHO/ATCC) database).

The following definitions were used:

1. Nosocomial (or healthcare-associated) MSSA and MRSA acquisition; was defined as isolation of MSSA and MRSA from any specimen including surveillance probes obtained from a surgical inpatient more than 48 h after admission who was previously identified as non-carrier (as determined by non-recovery of *S. aureus* from nasal swabs and/or open wound swabs of the same patient);
2. Nosocomial (or healthcare-associated) infection by MSSA and MRSA, respectively; was defined as the detection of MSSA and MRSA in a purulent specimen, superficial or deep soft-tissue lesion, skin abscesses, blood, sputum, or urine samples, obtained 48 h or more after admission. In addition, infection was only assumed, if at least two clinical symptoms of infection (e.g., fever, localized pain) associated with the respective site were present;
3. Surgical-site *S. aureus* infection; was defined as recovery of *S. aureus* from a superficial or deep surgical incision site, with or without drainage within 30 days after surgery.

Hospitalized surgical patients of the Obafemi Awolowo University Teaching Hospitals Complex in Ile-Ife and aged 01–90 years were included. Inclusion criteria were (i) hospitalization for a minimum period of 48 h, (ii) clinical evaluation for possible elective or emergency surgical intervention (irrespective of the ward to which the patient was initially admitted), (iii) antibiotic use after admission, (iv) and written (occasionally, if not literate, oral) informed consent to participate in the study. For pediatric patients, consent was sought from caregivers. Exclusion from the present study occurred if (i) hospitalization was less than 48 h, or medical records not accessible at the time of screening, (ii) clinical and laboratory evidence of *S. aureus* infection was documented at the time of admission, (iii) intermittent nasal discharge (e.g., catarrh, epistaxis), compromised nasal mucosal epithelium (e.g., cuts, abrasions), or a nasal device (e.g., endotracheal or nasogastric intubation, other devices e.g., oxygenation mask) was present, and (iv) convalescent patient(s) on discharge list. Surgical inpatients were recruited by a letter of invitation (in English).

### 4.3. Samples

Within the first 48 h after admission, swabs from the anterior nares were taken using a dry, sterile, cotton-tipped applicator (MicroPoint Diagnostics). If patients had open wounds or fractures at admission (i.e., open wounds from traffic accidents, gun shots, machetes, burns, other skin and soft tissue lesions such as superficial pressure ulcers or diabetic ulcers), these sites were also sampled. Subsequently, follow-up (peri-operative) specimens were obtained from various anatomical sites suspected of infection including skin abscesses and other symptomatic skin lesions, subcutaneous soft-tissue, deep soft-tissue, musculoskeletal tissue, peripheral blood, sputum, urine, or surgical incision sites. All wards were visited at least twice weekly for subsequent data collection and validation of nursing charts, drug prescription sheets, microbiology laboratory notes, etc. for all patients enrolled. All enrolled surgical inpatients considered eligible for surgical intervention also received standard peri-operative antibiotic prophylaxis, in accordance with prevailing anti-infective procedures within Obafemi Awolowo University Teaching Hospitals in Ile-Ife, at the time of the present study.

### 4.4. Microbiological Techniques

Clinical specimens and nasal swabs were cultivated according to standard procedures on Mueller–Hinton broth at 37 °C for 24 h, mannitol salt agar at 37 °C for 48 h, and on Columbia blood agar at 37 °C for 24 h, before molecular studies. *S. aureus* was presumably identified by catalase slide test, coagulase tube test, and Pastorex Staph-Plus test, performed according to the manufacturer’s protocol (Bio-rad, Marnes-la-Coquette, France) [2,52]. Antimicrobial susceptibility testing was achieved by the VITEK-2 Antimicrobial Susceptibility Testing automated systems, using the AST-P580 card according to the manufacturer’s specifications (bioMérieux, Marcy l’Etoile, France) and EUCAST clinical breakpoints (version 1.3) to define susceptibility. Species confirmation was achieved by Matrix-Assisted Laser Desorption Ionization Time-of-Flight mass spectrometry (MALDI Biotyper system, Bruker Daltonics, Bremen, Germany) and, additionally, by PCR targeting the *S. aureus nuc* gene as described elsewhere [53,54,55]. DNA was extracted from *S. aureus* cells using the QIA amp tissue kit (Qiagen, Hilden, Germany) by following the manufacturer’s recommendations. Methicillin resistance was confirmed by PCR targeting *mec*A and *mec*C, respectively [56,57].

### 4.5. Toxin Genes Detection

In accordance with standard *S. aureus* genotyping protocols, multiplex PCRs for detection of staphylococcal exotoxin genes including exfoliative toxin genes (*eta, etb*, and *etd*), epidermal differentiation inhibitor genes (*edinA, edinB*, and *edinC*), the staphylococcal pyrogenic toxin superantigen (PTSAg) genes including the toxic shock toxin 1 gene (*tst*) and enterotoxin/enterotoxin-like genes (*sea*, *seb*, *sec*, *sed*, *see*, *seg*, *seh*, *sei*, and *sej*), and *luk*F-PV and *luk*S-PV genes encoding PVL, were conducted for every *S. aureus* isolate as previously described [58,59,60].

### 4.6. Typing of S. aureus Isolates

For every *S. aureus* isolate, the polymorphic X-region of the *S. aureus* protein A gene (*spa*) was sequence-typed as previously described [61]. The “based upon repeat patterns” (BURP) algorithm of the StaphType software (Ridom GmbH, version 1.5, Münster, Ger-many) was applied, to cluster related *spa* types, using the default parameters described by Mellmann et al. [62]. Subtypes of the accessory gene regulator (*agr* I, II, III, and IV) were detected by multiplex PCR as previously described [63]. We performed multilocus sequence typing (MLST) for representative *spa* types in the present study [64]. MLST sequence types were clustered into groups as described by Mellmann et al. [62].

### 4.7. Statistical Approaches

The Student’s *t*-test, or F-test, was applied to test the significance of the difference between mean values, or variances, respectively. Comparison between groups and stratification analyses, were accomplished by chi-square test. The difference between expected and observed proportions of *S. aureus* infection among *S. aureus* carriers was tested using z-test. To obviate erroneous conclusions of significance, a continuity correction for cell frequencies less than 10 was implemented in (Mantel–Haenszel) chi-square. Where cell frequencies were appreciably low (≤2), Fisher’s exact results superseded chi-square-derived *p*-values. The utility function “StatCalc” in Epi Info^TM^ version 3.5.4 (Centers for Disease Control and Prevention, (CDC) Atlanta, USA) was utilized for sample size derivation and calculations. For exploratory comparison of patient characteristics, *p*-values were assessed for statistical significance on a case-by-case basis (at *p* < 0.05). For the hypothesis based on the chi-square test (implemented in the study design), a more stringent *p*-value (*p*≤ 0.01) determined significance of the outcome(s).

## 5. Conclusions

In contrast to widely varied epidemiology of *S. aureus* infection in industrialized settings, *S. aureus* more commonly was implicated in opportunistic SSTIs and bone and joint-related infections than in bacteremia, respiratory tract infection, or urinary tract infection, in the present study. Essentially, acute *S. aureus* infection developed during hospital stay (involving mostly MLST (lineages) ST1, ST7, ST15, ST88, or ST152) was 4.6-fold higher among *S. aureus* carriers; in particular, those admitted with recent bone or skin and soft tissue trauma, whereas, compared to the high detection rate of *hlg* (100%; 47/47) in infection-related *S. aureus* isolates, PVL-encoding genes were less frequent (12.7%; 6/47). This might reflect the mobility of PVL genes or lineage-specific PVL restrictions and might indirectly suggest a more pertinent contribution of HLG-encoding genes (which share bicomponent leukotoxicity with PVL [46]), to *S. aureus* infection. Together, these findings reflect the nosocomial burden of toxigenic, MDR *S. aureus* infection among surgical inpatients admitted with co-morbidities involving the integumentary or musculoskeletal system, who may benefit from strategies to block potentially virulent MDR *S. aureus* in an African country.

## Figures and Tables

**Table 1:** Baseline characteristics of the surgical inpatient cohort (*n* = 325).

Characteristics *	Male	Female	Total
Total number of patients enrolled	182	143	325
Age, mean ± SD	36.0 ± 18.6	37.4 ± 20.3	36.6 ± 19.4
Median (range)	35.6 (1–83)	30.4 (1–90)	35.8 (1–90)
LOS, mean days ± SD	33.9 ± 32.5	36.0 ± 40.3	34.8 ± 36.1
Specialty affiliation of patients:			
General Surgery	12 (6.6)	13 (9.1)	25 (7.7)
Cardiothoracic surgery	1 (0.5)	2 (1.4)	3 (1.0)
Abdominal surgery	9 (4.9)	18 (12.6)	27 (8.3)
Plastic surgery	25 (13.7)	5 (3.5)	30 (9.2)
Orthopedic surgery	119 (65.4)	77 (53.8)	196 (60.3)
Urology	7 (2.1)	2 (1.4)	9 (2.7)
Gynecology	-	13 (9.0)	13 (4.0)
Pediatric surgery	8 (4.4)	11 (7.7)	19 (5.8)
Unspecified type of surgery	1 (0.5)	2 (1.4)	3 (0.9)
No. of patients with *S. aureus* carriage within 48 h after admission	45 (24.7)	17 (11.9)	62 (19.0)
No. of patients with MRSA carriage	8 (4.4)	4 (2.8)	12 (3.7)
No. of patients with MSSA carriage	37 (20.3)	13 (9.0)	50 (15.4)
No. of patients with PVL-positive *S. aureus* carriage:	7 (3.8)	4 (2.8)	11 (3.4)
Of which PVL-positive MSSA carriage	5 (2.7)	3 (2.1)	8 (2.5)
Of which PVL-positive MRSA carriage	2 (1.1)	1 (0.7)	3 (0.9)
No. of patients without *S. aureus* carriage at screening	136 (74.7)	127 (88.8)	263 (80.9)
Total no. of patients with co-morbid conditions	153 (84.1)	129 (90.2)	279 (85.8)
McCabe score (at screening): Mean (Range)	1.0 (1.0–2.0)	1.1 (1.0–2.0)	1.1 (1.0–2.0)
No. patients receiving antibiotics without activity against MRSA	156 (85.7)	106 (74.1)	262 (80.6)
No. patients receiving antibiotic treatment active against MRSA	1 (0.5)	2 (1.4)	3 (1.0)
No. of patients who underwent surgical procedures	138 (75.8)	98 (68.5)	236 (72.6)
No. of patients with two or more surgical procedures	56 (30.7)	34 (23.7)	90 (27.7)
Surgical procedures (CDC classification)			
Clean	26 (12.7)	31 (22.8)	57 (16.7)
Clean-Contaminated	178 (87.3)	106 (77.9)	284 (83.3)
Emergency	18 (8.8)	15 (11.0)	33 (9.7)
Elective	186 (91.2)	122 (88.9)	308 (90.3)
Total number of surgical procedures	204 (59.8)	137 (40.2)	341 (100)
Incomplete medical records	9 (4.9)	5 (3.5)	14 (4.3)
Intravenous devices > 24 h	99 (54.4)	71 (49.6)	170 (52.3)
Urinary catheters > 24 h	52 (28.5)	41 (28.6)	93 (28.6)
Hemodialysis	1 (0.5)	0 (0.0)	1 (0.31)
Total no. of inpatient-days	6,137	5,155	11,292

* Data are no. (%) of patients, except indicated differently. (mean McCabe score (range): 1.1 (1.0–2.0). For all inpatients enrolled, an aggregate of 11,292 inpatient-days was accumulated during the period of the study.

**Table 3:** Risk factors for nosocomial *S. aureus* infection in nasal *S. aureus* carriers and nasal *S. aureus* non-carriers among surgical inpatients (*n* = 325).

Variable	*S. aureus* Carriers (*n* = 62)		*S. aureus* Non-Carriers (*n* = 263)		*p*-Value ^a^	Relative Prevalence (99% CI)
	*n*	*n* with Infection (% Carriers)	*n*	*n* with Infection (% Non-Carriers)		
Nosocomial *S. aureus* infection	62	27 (43.5)	263	20 (7.6)	0.0001	5.73 (2.93-11.13)
MRSA	12	3 (25.0)	313	4 (1.3)	0.0001^MH^	19.56 (3.18-120.2)
Hospitalized ≤ 12 months	15	7 (46.6)	53	7 (13.2)	0.002^MH^	3.53 (1.12-11.2)
Antibiotic therapy ≤ 12 months	6	4 (66.6)	11	4 (36.36)	0.122^MH^	1.83 (0.51-6.51)
Intravenous device ≤ 12 months	12	5 (41.6)	27	3 (11.1)	0.015^MH^	3.75 (0.71-19.63)
Surgery ≤ 12 months	9	3 (33.3)	24	5 (20.8)	0.231^MH^	1.60 (0.33-7.84)
LOS (This study) ≤ 3days	3	1 (33.3)	28	0 (0.0)	0.096^F^	Undefined
LOS ≤ 2 weeks	14	3 (21.4)	83	5 (6.0)	0.026^MH^	3.55 (0.63-20.02)
LOS ≤ 4 weeks	12	7 (58.3)	60	6 (10.0)	0.0001^MH^	5.83 (1.79-18.97)
LOS ≤ 8 weeks	19	8 (42.1)	50	3 (6.0)	0.0001^MH^	7.02 (1.42-34.75)
LOS ≤ 12 weeks	8	5 (62.5)	25	4 (16.0)	0.005^MH^	3.91 (0.98-15.45)
LOS > 12 weeks	6	3 (50.0)	17	2 (11.7)	0.088^F^	4.25 (0.57-31.66)
Antibiotic use (this study)	58	26 (44.8)	207	20 (9.6)	0.0001	4.64 (2.38-9.01)
Intravenous device (˃24 h)	24	9 (37.5)	64	4 (6.3)	0.0001^MH^	6.00 (1.45-24.82)
Urinary catheter (˃24 h)	2	1 (50.0)	8	0 (0.0)	0.200^F^	Undefined
Hemodialysis	1	0 (0.0)	0	0	-	Undefined
Surgery (this study)	53	22 (41.5)	184	15 (8.2)	0.0001	5.09 (2.37-10.92)
Emergency	12	9 (75.0)	21	2 (9.5)	0.0001^MH^	7.87 (1.32-46.93)
Elective	41	13 (31.7)	163	13 (7.9)	0.0001	3.98 (1.61-9.82)
≥ 2 surgeries (This study)	29	16 (55.2)	61	10 (16.4)	0.0001	3.37 (1.42-7.95)
Comorbidities	49	24 (48.9)	176	20 (11.4)	0.0001	4.31 (2.23-8.34)
Bone disease.	17	5 (29.4)	55	4 (7.3)	0.008^MH^	4.04 (0.84-19.5)
Skin and soft-tissue disease	7	3 (42.8)	28	4 (14.3)	0.047^MH^	3.00 (0.58-15.45)
Bone, skin and soft-tissue disease	19	13 (68.4)	38	9 (23.7)	0.001^MH^	2.89 (1.23-6.76)
Cardiovascular disease	2	2 (100)	17	2 (11.7)	0.035^F^	8.50 (1.54-47.04)
Pulmonary disease	1	0 (0.0)	4	2 (50.0)	0.600^F^	2.0 (0.55-7.25)
Genitourinary disease	1	0 (0.0)	15	1 (6.6)	0.125^F^	15.0 (1.25-180.6)
Neurological disease	0	0 (0.0)	8	2 (25.0)	-	Undefined
Diabetes mellitus	1	1 (100)	8	1 (12.5)	0.222^F^	8.00 (0.72-89.03)
Total number of patient-days	2,529	1,441 (56.9)	8,763	996 (11.4)	0.0001	5.01 (4.58-5.48)

^a^ Uncorrected chi-square *p*-values were derived (*p* ≤ 0.01). For uniform reporting of *p*-values, and for continuity corrections (cell frequencies ≤ 10), Mantel–Haenszel-corrected chi-square *p*-values superseded crude uncorrected chi-square *p*-values, Fisher exact *p*-values were derived for cell frequencies ≤ 2. All *p*-values are one-tailed. Degree of freedom = 1. Dashed lines indicate any row or column total = 0, for which no statistical- or *p*-values are derivable. Undefined risk ratios are indicated. MRSA—methicillin-resistant *S. aureus*, F—Fisher exact *p*-value, MH—Mantel–Haenszel, LOS—length of stay.

## Data Availability

The data analyzed in the present study are available within the article and Supplementary Materials section.

## Supplementary Materials

The following supporting information can be downloaded at: https://www.mdpi.com/article/10.3390/antibiotics11101372/s1, Table S1: Occurrence of *S. aureus* infection among *S. aureus* nasal-carriers and non-carriers, stratified according to indication for admission. Table S2: Characteristics of inpatients with, or without any type of nosocomial *S. aureus* infection. Figure S1; Surgical procedures related to, or not related to *S. aureus* infection.
