# Supplementary material for: Local Epidemiology of Nosocomial Staphylococcus aureus Infection in a Nigerian University Teaching Hospital
Source: Antibiotics (Basel). 2022 Oct 7;11(10):1372. doi: 10.3390/antibiotics11101372 (PMC9598115; doi:10.3390/antibiotics11101372)
Supplement: Supplementary file 1 [file antibiotics-11-01372-s001.zip › antibiotics-1931272-suppl.pdf]

antibiotics

MDPI

Table S1. Occurrence of *S. aureus* infection among *S. aureus* nasal-carriers and non-carriers, stratified according to indication for admission.

Characteristic		Open Fractures (98/325; 30.2%)		Closed Fractures (42/325; 12.9%)		Failure of Implants (7/325; 2.1%)		Open Wounds not Fractures e.g. Burns (n=9), Gun Shots (n=7), Cutaneous Ulcers (n=4) (20/325; 6.2%)		Bone Disease not Fractures (14/325; 4.3%)		[#]Others (132/325; 40.6%)	
		Carriers (%)	Non-Carriers (%)	Carriers (%)	Non-Carriers (%)	Carriers (%)	Non-Carriers (%)	Carriers (%)	Non-carriers (%)	Carriers (%)	Non-carriers (%)	Carriers (%)	Non-Carriers (%)
No.of patients		21 (21.4%)	77 (78.5%)	15 (35.7%)	27 (64.2%)	3 (42.8%)	4 (57.1%)	5 (25.0%)	15 (75.0%)	4 (28.5%)	10 (71.4%)	14 (10.6%)	118 (89.4%)
S. aureus-contaminated wounds		19	16	10	4	2	1	3	2	3	1	6	2
S. aureus infection, n (%):		11 (52.4%)	6 (7.80%)	3 (20.0%)	3 (11.1%)	0 (0.0%)	2 (50.0%)	5 (100%)	4 (26.6%)	3 (75.0%)	2 (20.0%)	5 (35.7%)	3 (2.5%)
Surgical site infection, n (%)		4 (36.4%)	4 (66.6%)	1 (33.3%)	1 (33.3%)	0 (0.0%)	0 (0.0%)	4 (80.0%)	1 (25.0%)	0 (0.0%)	0 (0.0%)	4 (80.0%)	0 (0.0%)
MSSA (*spa* type: n; clinical entity (n))		t091: 10; SSI (4), AO (3), fasciitis (1), CAUTI (2)	t091: 4; AO (2), SSI (2). t355: 1; SSI. t127: 1; SSI	t091: 3; SSI (1), AO (1), CA (1)	t091: 1; CAUTI	NA	t084: 2; peri-implant bone infections	t091: 4; SSI	t311: 1; CA. t7762: 1; CA. t084: 2; DSI (1), bacteremia (1)	t127: 1; AO. t2724: 1; pyomyositis	t091: 1; AO	t091: 5; SSI (4), SSTI (1)	t2731: 1; pneumonia. t085: 1; CA. t064: 1; pneumonia
MRSA (*spa* type: n; clinical entity (n))		t786: 1; acute septic arthritis	t091: 1; DSI.	ND	t008: 1; DSI. t037:1; CO	NA	ND	t786: 1; CA	ND	t355: 1; abscess	t355: 1; pyomyositis	ND	ND
No. isolates with *hlg* genes		11	6	3	3	NA	2	5	4	3	2	5	3
Classical PTSAg genes (*spa* type)	MSSA	ND	*sea* (t127)	ND	*sea* (t091)	NA	*sea* (t084; 2)	ND	*sea* (t084:2)	ND	ND	ND	*sea*, *seb* (t085), *sed* (t064)
	MRSA	ND	ND	NA	*sea*, *seb* (t008)	NA	NA	ND	NA	ND	ND	NA	NA
Other PTSAg genes (*spa* type)	MSSA	ND	*seh* (t127)	ND	ND	NA	ND	ND	*seg-sei* (t311), *seh* (t7762)	*seg-sei* (t2724), *seh* (t127),	ND	ND	*seg-sei* (t2731)
	MRSA	ND	ND	NA	NA	NA	NA	ND	NA	*sei* (t355)	ND	NA	NA
edin genes (n)		ND	ND	ND	ND	NA	ND	ND	*edin*A (t311)	*edin*A (t2724)	ND	ND	ND
lukSF-PV (PVL-toxin genes) (n)		ND	ND	ND	*lukSF*-PV (t091)	NA	*lukSF*-PV (t084:	ND	*lukSF*-PV (t084) DSI	*lukSF*-PV (t355)	*lukSF*-PV (t355)	ND	ND

						2)						
MLST STs	7, 88	7, 152, 1	7	7, 8, 241	NA	15	7, 88	15, 1, 15	1, 1, 152	7, 152	7	5, 15, 8
agr type	III; IV	III	III	III	NA	II, IV	III; IV	II; IV; II	IV; II; III	III	III	III, II, II

MSSA, methicillin susceptible *Staphylococcus aureus*; MRSA, methicillin resistant *Staphylococcus aureus; hlg*, gamma hemolysin genes; PTSAg, pyrogenic toxin superantigens; *sea*, staphylococcal enterotoxin A gene; *seb*, staphylococcal enterotoxin B gene; *sec*, staphylococcal enterotoxin C gene; *sed*, staphylococcal enterotoxin D gene; *seg,* staphylococcal enterotoxin G gene; *seh*, staphylococcal enterotoxin H gene; *sei*, staphylococcal enterotoxin I gene; *sej*, staphylococcal enterotoxin J gene; *edin*, epidermal differentiation inhibitor genes; *lukSF*-PV, PVL toxin-encoding genes; NA, not applicable; ND, not detected. [#]Respiratory disease (n = 5); abdominal disorders (n = 36); neoplastic conditions (n = 31); genitourinary disease (n = 23); congenital defects (n = 3); lymphatico-venous disorders (n = 4); unknown (n = 30). Among 1, 4, and 7 patients respectively admitted with cerebrovascular, cardiovascular and neurological indications, neither *S. aureus* carriage nor infection was observed (not shown). AO, acute osteomyelitis; CO, chronic osteomyelitis; CA, cutaneous abscess; CAUTI, catheter-associated urinary tract infection; SSI, superficial surgical site infection; DSI, deep surgical site infection; MLST, multilocus sequence type (in order of the first appearance of *spa* type); ST, sequence type; *agr*, accessory gene regulator (in order of the first appearance of *spa* type).

Table S2. Characteristics of inpatients with, or without any type of nosocomial *S. aureus* infection.

**Variable**	[a]**Patients with *S. aureus* Infection**			[b]**Patients without *S. aureus* Infection**			***p*-Value ***	**Odds Ratio (Exact 95% Confidence Limits)**
	Male (n=31)	Female (n=16)	Total (n=47)	Male (n=152)	Female (n=126)	Total (n=278)		
Age: 1- 9 years	2 (6.45)	3 (18.75)	5 (10.6)	14 (9.21)	7 (5.55)	21 (7.55)	0.471	1.46 (0.41 - 4.26)
Age: 10-19 years	2 (6.45)	2(12.5)	4 (8.51))	7 (4.60)	7 (5.55)	14 (5.04)	0.335	1.75 (0.40 - 5.93)
Age: 20-29 years	8 (25.80)	1 (6.25)	9 (19.14)	33 (21.71)	22 (17.46)	55 (19.78)	0.919	0.96 (0.39 - 2.18)
Age: 30-39 years	11 (35.48)	4 (13.33)	15 (31.91)	23 (15.13)	33 (26.19)	56 (20.14)	0.071	1.86 (0.87 - 3.82)
Age: 0-39 years	23 (74.2)	10 (62.5)	33 (70.2)	77 (50.6)	69 (54.7)	146 (52.5)	0.024	2.13 (1.05 – 4.50)
Age: 40-49 years	5 (16.13)	2 (12.50)	7 (14.89)	27 (17.76)	16 (12.69)	43 (15.46)	0.919	0.96 (0.34 - 2.35)
Age: 50-59 years	2 (6.45)	0 (0.00)	2 (4.25)	15 (9.86)	10 (7.93)	25 (8.99)	0.395[F]	0.45 (0.05 - 1.92)
Age: 60-69 years	0 (0.00)	1 (6.25)	1 (2.12)	14 (17.10)	7 (19.04)	50 (17.98)	0.005	0.10 (0.00 - 0.61)
Age: 70-79 years	0 (0.00)	1 (6.25)	1 (2.12)	7 (4.60)	14 (11.11)	21 (7.55)	0.221[F]	0.26 (0.01 - 1.72)
Age: 40-79 years	7 (22.58)	4 (25.00)	11 (23.4)	63 (41.44)	47 (37.3)	110 (39.56)	0.033	0.47 (0.21 – 0.99)
Age: 80-89 years	0 (0.00)	0 (0.00)	0 (0.00)	5 (3.28)	2 (1.58)	7 (2.52)	0.598[F]	0.00 (0.00 - 5.08)
Age: 90-99 years	0 (0.00)	0 (0.00)	0 (0.00)	0 (0.00)	1 (0.79)	1 (0.36)	1.000[F]	0.00 (0.00 - 230.68)
[#]Mean age ± SD	29.6 ± 11.9	24.06 ± 21.9	28.9 ± 16.82	38.2 ± 19.7	40.0 ± 19.7	39.0 ± 20.4	0.0008	-
[##]Median age ± SE (IQR)	31.2 ± 2.68 (25.5 - 36.5)	30.5 ± 6.86 (13.5 - 40.5)	31.0 ± 3.07 (23.5 - 39.5)	38.5 ± 2.01 (26.5 - 53.5)	37.25 ± 2.21 (27.5 - 54.5)	37.1 ± 1.54 (26.5 - 54.5)	-	-
Variance	141.99	480.28	283.03	391.29	391.92	418.85	0.1089	-
Recent hospital stay	7 (22.58)	6 (37.50)	13 (27.65)	20 (13.15)	10 (7.93)	30 (10.79)	0.0002	3.64 (1.60 - 7.96)
Recent intravenous device	4 (12.90)	4 (25.00)	8 (17.02)	13 (8.55)	5 (3.96)	18 (6.47)	0.014	2.96 (1.04 - 7.74)
Recent surgery	4 (12.90)	4 (25.00)	8 (17.02)	8 (5.26)	6 (4.76)	14 (5.03)	0.002	3.87 (1.31 - 10.60)
Recent antibiotic use	5 (16.12)	4 (25.00)	9 (19.14)	9 (5.92)	6 (4.76)	15 (5.39)	0.001	4.15 (1.48 - 10.91)
Carriage (anterior nares)	20 (64.50)	7 (43.75)	27 (57.44)	25 (16.44)	10 (7.93)	35 (12.58)	0.0001	9.37 (4.49 - 19.53)

**Variable**	**[a]Patients with *S. aureus* Infection**			**[b]Patients without *S. aureus* Infection**			***p*-Value ***	**Odds Ratio (Exact 95% Confidence Limits)**
	Male (n=31)	Female (n=16)	Total (n=47)	Male (n=152)	Female (n=126)	Total (n=278)		
MRSA	3 (9.67)	0 (0.00)	3 (6.38)	4 (2.63)	4 (3.17)	8 (2.87)	0.219	2.30 (0.38 - 10.03)
PVL genes	2 (6.45)	0(0.00)	2 (4.25)	5 (3.28)	4 (3.17)	9 (3.23)	0.663[F]	1.33 (0.14 - 6.72)
MRSA+PVL genes	1 (3.22)	0 (0.00)	1 (2.12)	0 (0.00)	3 (2.38)	3 (1.08)	0.466[F]	1.99 (0.04 - 25.37)
MSSA	18 (58.06)	6 (37.50)	24 (51.06)	19 (12.5)	6 (4.76)	25 (8.99)	0.0001	10.56 (4.90 - 22.59)
MSSA+PVL genes	1 (3.22)	0 (0.0)	1 (2.12)	5 (3.28)	1 (0.79)	6 (2.15)	1.000[F]	0.99 (0.02 - 8.42)
PSSA	0 (0.00	0 (0.00)	0 (0.00)	3 (1.97)	0 (0.00)	3 (1.07)	1.000[F]	0.00 (0.00 - 31.71)
Antibiotic use (this study)	29 (93.55)	16 (100.00)	46 (97.87)	126 (82.89)	91 (72.22)	217 (78.05)	0.001	12.93 (2.10 - 530.12)
Surgery: Yes	24 (77.42)	13 (81.25)	37 (78.72)	114 (75.00)	85 (67.46)	199 (71.58)	0.309	1.47 (0.67 - 3.47)
Surgery: No	7 (22.58)	3 (18.75)	10 (21.2)	35 (23.02)	40 (31.74)	75 (26.97)	0.411	0.73 (0.31 – 1.60)
Emergency	6 (19.35)	5 (31.25)	11 (23.40)	12 (7.89)	10 (7.93) \	22 (7.91) \	0.007	3.06 (1.13 - 7.63)
Elective	18 (58.06)	8 (50.0)	26 (55.31)	101 (66.44)	76 (60.31)	177 (54.46)	0.274	0.71 (0.36 – 1.40)
Two or more surgeries	17 (54.83)	9 (56.25)	26 (55.31)	37 (24.34)	23 (18.25)	60 (21.58)	0.0001	4.50 (2.25 - 9.01)
Clean surgery	17 (54.83)	3 (18.75)	20 (42.55)	52 (34.2)	44 (34.92)	96 (34.53)	0.288	1.40 (0.71 - 2.75)
Clean-contaminated surgery	7 (22.58)	10 (62.5)	17 (36.17)	41 (26.97)	35 (27.77)	76 (27.33)	0.215	1.51 (0.73 - 3.01)
General surgery	2 (6.45)	1 (6.2)	3 (6.4)	9 (5.9)	13 (10.3)	22 (7.9)	0.715	0.79 (0.15 - 2.81)
Cardiothoracic surgery	0 (0.00)	0 (0.00)	0 (0.00)	1 (0.6)	2 (1.6)	3 (1.1)	1.000[F]	0.00 (0.00 - 13.42)
Abdominal surgery	0 (0.00)	0 (0.00)	0 (0.00)	8 (5.2)	19 (15.1)	27 (9.7)	0.020[F]	0.00 (0.00 - 0.82)
Plastic surgery	5 (16.13)	1 (6.2)	6 (12.7)	19 (12.5)	5 (3.9)	24 (8.6)	0.365	1.55 (0.49 - 4.20)
Orthopedic surgery	18 (58.06)	7 (43.7)	25 (53.2)	101(67.1)	71 (57.1)	172 (61.8)	0.260	0.70 (0.36 - 1.38)
Urology	1 (3.22)	0 (0.00)	1 (2.1)	6 (3.9)	2 (1.6)	8 (2.8)	1.000[F]	0.73 (0.02 - 5.70)
Gynecology	-	1 (6.2)	1 (2.1)	-	12 (9.5)	12 (4.3)	0.701[F]	0.48 (0.01 - 3.41)
Pediatrics	4 (12.90)	5 (31.2)	9 (19.1)	4 (2.63)	6 (4.76)	10 (3.6)	0.0002	6.35 (2.12 - 18.49)

**Variable**	[a]**Patients with *S. aureus* Infection**			[b]**Patients without *S. aureus* Infection**			***p*-Value ***	**Odds Ratio (Exact 95% Confidence Limits)**
	Male (n=31)	Female (n=16)	Total (n=47)	Male (n=152)	Female (n=126)	Total (n=278)		
Internal medicine	1 (3.22)	1 (6.2)	2 (4.2)	0 (0.00)	1 (0.8)	1 (0.3)	0.055[F]	12.31 (0.62 - 728.79)
Bone disease	20 (64.51)	10 (62,50)	30 (63.83)	47 (30.92)	39 (30.95)	86 (30.93)	0.0001	3.94 (1.97 - 8.02)
Skin and soft tissue disease	6 (19.35)	4 (25.00)	10 (21.27)	16 (10.52)	10 (7.93)	26 (9.35)	0.015	2.62 (1.04 - 6.16)
Cardiovascular disease	1 (3.22)	3 (18.75)	4 (8.51)	7 (4.60)	8 (6.34)	15 (5.39)	0.399	1.63 (0.38 - 5.44)
Bone, and skin and soft tissue disease	13 (41.93)	9 (56.25)	22 (46.80)	20 (13.15)	15 (11.90)	35 (12.58)	0.0001	6.11 (2.93 - 12.59)
Pulmonary disease	1 (3.22)	1 (6.25)	2 (4.25)	2 (1.31)	1 (0.79)	3 (1.08)	0.153[F]	4.07 (0.33 - 36.36)
Lymphoid disorders	0 (0.00)	0 (0.00)	0 (0.00)	2 (1.31)	1 (0.79)	3 (1.08)	1.000[F]	0.00 (0.00 - 14.47)
Genitourinary disease	0 (0.00)	1 (6.25)	1 (2.12)	6 (3.94)	5 (3.96)	11 (3.95)	1.000[F]	0.53 (0.01 - 3.80)
Gastrointestinal tract disease	0 (0.00)	0 (0.00)	0 (0.00)	10 (6.57)	13 (10.31)	23 (8.27)	0.057[F]	0.00 (0.00 - 0.99)
Neurological deficit (focal)	1 (3.22)	1 (6.25)	2 (4.25)	7 (4.60)	1 (0.79)	8 (2.80)	0.642[F]	1.58 (0.15 - 7.85)
Diabetes/metabolic disease	1 (3.22)	1 (6.25)	2 (4.25)	2 (1.31)	5 (3.96)	9 (3.23)	0.663[F]	1.33 (0.14 - 6.72)
Intravenous device > 24 h	22 (70.96)	13 (81.25)	35 (74.46)	75 (49.34)	55 (43.65)	130 (46.76)	0.0004	3.32 (1.60 - 7.31)
Urinary catheter > 24 h	6 (19.35)	11 (68.75)	17 (36.17)	26 (17.10)	15 (11.90)	41 (14.74)	0.0003	3.28 (1.54 - 6.77)
Average time to infection, d median (IQR)	15.5 (7.5 - 42.5)	23.75 (7.5 - 42.5)	17.5 (6.5 - 42.5)	NA	NA	NA	NA	NA
LOS d mean ± SD	42.03 ± 29.68	69.43 ± 63.20	51.36 ± 45.96	31.53 ± 32.65	32.23± 32.68	31.85 ± 32.67	0.007	-
LOS median (IQR.)	29.5 (21.5 – 59.5)	47.5 (24.5 - 86.5)	35.5 (21.5 – 69.5)	21.5 (8.5 – 46.5)	22.5 (7.5 – 38.25)	24.5 (7.25 - 43.15)	-	-
LOS variance	880.9	3,994.24	2,112.3	1,066.0	1,067.9	1,067.3	0.0006	-
Total no. of patient days	1,318 (54.08)	1,119 (45.9)	2,437 (21.58)	4,830 (54.54)	4,025 (45.45)	8,855 (78.41%)	-	-

*Chi-square *p*-value (two-tailed), derived according to chi-square test for the significance of the difference between uncorrelated proportions, F-Fisher exact *p*-value (two-tailed): significance implied at $p < 0.05$. IQR, interquartile range; LOS, length of stay; d, day. [#]Mean, ± standard error of the mean; [##]Median, ± standard error of the median; NA, Not applicable. [a]Three patients with *S. aureus* infection whose age could not be determined, were included in the final analyses. [b]Fourteen patients without *S. aureus* infection, whose age could not be ascertained were included in the final

analyses. MSSA, methicillin-susceptible *S. aureus*; PSSA, penicillin-susceptible *S. aureus*; MRSA, methicillin-resistant *S. aureus;* PVL, Panton-Valentine Leukocidin.

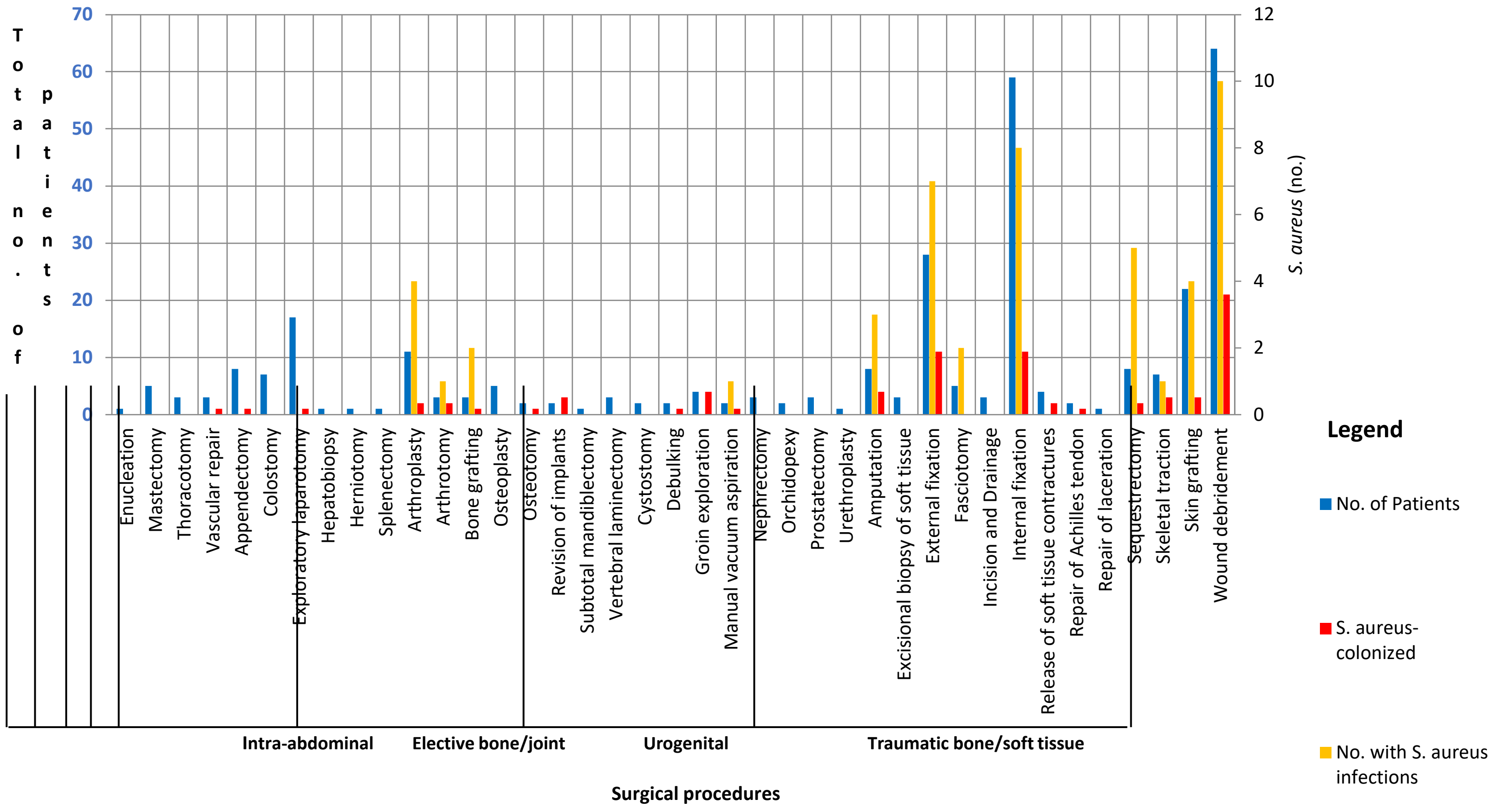

Figure S1. Surgical procedures related to or, not related to *S. aureus* infection in this study; Total no. of patients (primary Y-axis), surgical procedures (X-axis), *S. aureus* (no.) (secondary Y-axis).
